# Current immunological and molecular tools for leptospirosis: diagnostics, vaccine design, and biomarkers for predicting severity

**DOI:** 10.1186/s12941-014-0060-2

**Published:** 2015-01-16

**Authors:** Senaka Rajapakse, Chaturaka Rodrigo, Shiroma M Handunnetti, Sumadhya Deepika Fernando

**Affiliations:** Department of Clinical Medicine, Faculty of Medicine, University of Colombo, 25, Kynsey Road, Colombo, 08 Sri Lanka; Institute of Biochemistry, Molecular Biology and Biotechnology, University of Colombo, Colombo, Sri Lanka; Department of Parasitology, Faculty of Medicine, University of Colombo, Colombo, Sri Lanka

**Keywords:** Leptospirosis, Vaccine, Biomarkers, Diagnosis

## Abstract

Leptospirosis is a zoonotic spirochaetal illness that is endemic in many tropical countries. The research base on leptospirosis is not as strong as other tropical infections such as malaria. However, it is a lethal infection that can attack many vital organs in its severe form, leading to multi-organ dysfunction syndrome and death. There are many gaps in knowledge regarding the pathophysiology of leptospirosis and the role of host immunity in causing symptoms. This hinders essential steps in combating disease, such as developing a potential vaccine. Another major problem with leptospirosis is the lack of an easy to perform, accurate diagnostic tests. Many clinicians in resource limited settings resort to clinical judgment in diagnosing leptospirosis. This is unfortunate, as many other diseases such as dengue, hanta virus, rickettsial infections, and even severe bacterial sepsis, can mimic leptospirosis. Another interesting problem is the prediction of disease severity at the onset of the illness. The majority of patients recover from leptospirosis with only a mild febrile illness, while a few others have severe illness with multi-organ failure. Clinical features are poor predictors of potential severity of infection, and therefore the search is on for potential biomarkers that can serve as early warnings for severe disease. This review concentrates on these three important aspects of this neglected tropical disease: diagnostics, developing a vaccine, and potential biomarkers to predict disease severity.

## Introduction

Leptospirosis is a zoonotic disease caused by spirochaetes of the genus *Leptospira*. The disease results in high morbidity and considerable mortality in areas of high prevalence [1]. It is estimated that around 10,000 cases of severe leptospirosis are hospitalized annually worldwide [2]. The disease is endemic in areas with high rainfall, close human contact with livestock, poor sanitation and workplace exposure to the organism [3]. There are currently 14 identified potentially pathogenic species of leptospira (9 definite and 5 intermediate). Any mammal has the potential to be the reservoir for the organism, but it is predominantly rodents who play a role in transmitting infection to humans. The organisms can be transferred to humans through contact with body fluids and urine of infected animals, with entry of the organisms occurring through mucosal surfaces or breached skin [2].

Primarily manifesting as an acute febrile illness, severe forms of leptospirosis affect multiple organ systems, resulting in acute kidney injury, pulmonary haemorrhage, hepatitis, myocarditis, disseminated intravascular coagulation, and meningo-encephalitis. The case fatality rate in severe leptospirosis can exceed 40% [4]. It is postulated that severe disease is driven largely by the host immunological response rather than the pathogen’s virulence. There are a multitude of unresolved, practically relevant areas on this illness that need to be addressed by further research. In this review, we focus on three important areas, i.e., diagnostics, vaccine development, and identification of biomarkers of disease severity.

## Methods

A MEDLINE search was performed for articles with the keywords ‘leptospirosis’ OR ‘leptospira’ OR ‘Weil’s’ OR ‘Weil’ in title or abstract. The search was restricted to articles published in English within the last 15 years (1998 – September 2013) as they would contain more recent data. The search was also restricted to studies reporting on humans (not animals). There were 1442 abstracts in the original search with these restrictions. The software Endnote X3 was used to filter articles. Bibliographies of cited literature were also searched. All abstracts were read through independently by the three authors, and relevant papers were identified for review of the full papers. Related papers were also included. We reviewed 159 selected full papers.

These sources were screened for a well described methodology, accurate statistical analysis and an adequate sample size where relevant. Data sources included reviews published in core clinical journals, cohort studies, interventional studies, case control studies, cross sectional analysis and epidemiological data. Suitable data was available in 49 papers from the initially selected 159.

### Leptospirosis: diagnostic issues

Laboratory diagnosis of leptospirosis remains a challenge. There are many diagnostic tests for leptospirosis. These can be broadly divided according to their methodology into: a) methods demonstrating the organism in culture or clinical specimens, b) immunological methods, and c) genomic methods.

#### Direct demonstration of organisms

The simplest diagnostic procedure is demonstrating the organism in urine or blood with dark ground microscopy (DGM). However, the sensitivity and specificity is questionable, despite the low cost. In addition, ideal diagnostic conditions with DGM require the specimen to be prepared from culture, which is very difficult to achieve since the organism is fastidious. Chandrasekaran et al. [5] compared the usefulness of DGM vs. IgM ELISA, and concluded that DGM had high positivity in patients with clinically suspected leptospirosis compared to ELISA (95.5% vs. 64.7%). The positivity of DGM diminished, and that of ELISA increased (though still DGM had higher positivity) with the duration of infection. Comparison was not made against a gold standard in this case, and the study simply compared positivity with the two tests in clinically suspected cases. In another study of 297 samples, sensitivity and specificity of DGM was around 60% [6].

#### Immunological diagnostics

The immunological reference standard for diagnosis of leptospirosis is the microscopic agglutination test (MAT). However, this test involves the cumbersome procedure of reacting the patient’s serum with different panels of live leptospira antigens. MAT is not specific for IgM, and detects both IgM and IgG, and may not be able to differentiate acute from previous infection. Furthermore, there is little evidence on how long IgG antibodies persist in blood after acute infection. Thus ideally, the test requires two samples (acute and convalescent) for confirmation. In a clinical setting where rapid decision making is necessary, MAT is not be the ideal test to go by for diagnostic confirmation.

Other immunological tests available include IgM ELISA, microcapsule agglutination test, Lepto-dipstick, Lepto Dri Dot, and Leptocheck-WB test. These allow rapid diagnosis, and are simpler to perform than MAT. Still, all these tests can be negative in early leptospirosis as it takes time for antibodies to form. The sensitivities and specificities of the tests vary depending on the antibodies present and the leptospira antigen used. For example Boonyod and colleagues [7] demonstrated that a rapid diagnostic test using a dipstick for the outer membrane protein (OMP) LipL32, which is expressed in pathogenic leptospira, had good sensitivity and specificity to MAT (100% and 98.3% respectively). The suitability of this antigen for pathogenic leptospira diagnosis has been independently confirmed by others [8-10]. A group of investigators in Brazil assessed the use of *Leptospira* immunoglobulin (Lig)-like proteins as antigens to react with IgM antibodies in patient’s sera in an immunoblot assay. This had a sensitivity of 81% during the first 7 days of illness. Neves and colleagues [11] identified two proteins, namely Lp29 and Lp49, which were reactive with sera of patients during an outbreak in Brazil. These proteins were identified after screening the *L. interrogans* genome for potential sequences that code for outer membrane proteins. The IgM for these proteins were detected in sera of patients in both acute and convalescent phases, and IgM against Lp29 was detected when the MAT was negative in the acute phase of illness. However, it was not confirmed whether these proteins were present in all pathogenic serovars of leptospirosis. An IgM immunoblot test against antigens of several leptospira serovars prevalent in Thailand yielded positive results, with a sensitivity of 88% during the first three days since onset of symptoms (corresponding MAT sensitivity in this early sample was just 2%). ELISA assays based on recombinant products of OMPs are developed for locally circulating virulent organisms. Whether they would be useful outside a particular geographical area is doubtful. In a large scale study in Andaman Islands, researchers developed an IgM ELISA study for two OMPs (OmpL1 and LipL41) of locally prevalent virulent leptospira serovars that caused severe pulmonary leptospirosis [12]. The test had sensitivities and specificities ranging between 80-90% compared against MAT but may not be universally applicable for different serovars that are prevalent in other areas. Senthilkumar et al. [13], in an attempt to develop a rapid diagnostic method, assessed recombinant LipL41 protein as an antigen to be used in latex agglutination test (LAT) and flow through assays. The protein was conserved among all pathogenic species of leptospirosis. Both tests took less than 5 minutes to complete and had good sensitivities and specificities when compared against MAT (sensitivity and specificity 89.7% and 90.4% respectively for LAT, 77% and 89% for flow through assay). Other recombinant conserved proteins of pathogenic leptospira that have yielded good results in immunological diagnostics include: rligA [14], Hap1/lipL32 [15], and rLoa22 [16].

Another interesting aspect to leptospirosis diagnosis by immunological methods was adopted by Lin et al. [17]. They considered five immunodominant epitopes of three OMPs of pathogenic leptospira (OmpL1, LipL21, and LipL32) and constructed a synthetic gene (rlmp). The purified protein product of this gene was used as an antigen to react with patient sera (with both IgM and IgG antibodies) of confirmed leptospirosis patients in an ELISA test. The results were encouraging with no cross reactions and false positives in control groups, and detecting all MAT positive leptospirosis with the new test. In a similar experiment, Sun et al. [18] created a recombinant fusion protein of the same antigens that was later used in an IgM ELISA for early diagnosis of leptospirosis. The ELISA with the recombinant protein yielded better results (>95% sensitivity in a sample of 493 leptospirosis patients) than ELISA tests using each of the individual antigens. Additionally they demonstrated that this antigen did not cross react with sera of patients with non-leptospirosis fevers, such as dengue and typhus.

Finally, a recently published meta-analysis of ELISA diagnostic tests for leptospirosis holds that they have a fairly good sensitivity and specificity (77% and 91% respectively; area under the curve 0.964). The drawbacks were the heterogeneity among the tests and the lower yield in the initial phase of the illness [19]. This remains the main problem with IgM ELISA tests for leptospirosis, i.e., heterogeneity between different antigens used for testing essentially affects sensitivity among different strains of the organism.

While being less sophisticated and time consuming than the MAT, ELISA tests also need considerable laboratory support. Rapid diagnostic tests (RDT) are an alternative for on-field diagnosis with minimal laboratory support. Goris and colleagues [20] evaluated three commercially available RDTs (LeptoTek Dri Dot, LeptoTek Lateral Flow, and Leptocheck-WB) against the MAT and ELISA test results for the same samples. All three tests had sensitivity of more than 75% and specificity of at least 95%. However in order to obtain a better sensitivity, at least two samples had to be tested per patient (sensitivity for single samples ranged from 51-69%). It was concluded that RDTs alone cannot be relied upon to diagnose leptospirosis, especially in the earlier stages of the illness.

However, all these comparisons of different immunological diagnostic tests need a gold standard for a valid comparison. Until recently this gold standard was MAT (or culture which has low sensitivity as the organism is fastidious). Unfortunately, the MAT is in itself an imperfect gold standard, which makes the sensitivities and specificities of other tests judged against it less reliable and hence has to be interpreted with caution (see below) [21].

#### Genomic diagnostics

Genomic diagnostic tests have the advantage of being positive early in disease, but have the disadvantages of limited availability and high cost. There are several diagnostic techniques that can be employed in the genomic diagnosis. These are outlined below.

Polymerase chain reaction (PCR): Involves amplifying DNA sequences specific to the organism, using primers. Provided the sequence amplified is specific to the pathogen, this method has the potential to be 100% specific. Gravekamp et al. [22] developed two groups of primers (G1 & G2 and B64 I & B64 II) that were capable of diagnosing all genospecies of leptospira known upto the year 2003, and these had been used heavily in studies that required specific diagnosis of leptospirosis. De Abreu Fonseca et al. [23] compared the sensitivity and specificity of PCR against that of MAT and IgM ELISA in 124 serum samples (60 with confirmed leptospirosis). The specificity of PCR was 100% but the sensitivity varied between 44-62% with less sensitivity for samples collected later on in the infection. The sensitivities for MAT ranged between 69 -95% and increased with duration since infection (specificity of MAT ranged between 90 – 100%). Combination of PCR and ELISA increased the sensitivity to 93-95% during first week of infection. Similar findings have been demonstrated by other authors as well [24].

Arbitrarily primed PCR: This technique uses an arbitrary primer to amplify segments of DNA which on gel electrophoresis should produce a specific pattern of bands that is species specific. However, even within the same species, researchers have shown that arbitrary primed DNA banding patterns can differ.

Nucleic acid probes: This is a very specific technique that allows diagnosis of infection at a very early stage. Provided the probe is a specific one, it will enable species differentiation.

Restriction enzyme analysis (REA): Cleaving purified dsDNA of leptospira by restriction enzymes gives a specific DNA fingerprint when run on gel electrophoresis. Recognizing this pattern will enable to identify members of same species with same restriction sites. While this can be used as a diagnostic technique, application of this has also enabled to further genetically classify subspecies or identify new species that were previously thought to be a single species.

Random amplified polymorphic DNA fingerprinting (RAPD): This involves combination of arbitrary primer use and PCR to generate a unique pattern of genomic bands that is specific at species level. This technique has enabled rapid differentiation between different species but has the disadvantage of needing pure cultures to extract DNA.

Pulsed field gel electrophoresis: This is a technically cumbersome procedure of generating larger genomic fragments by restriction enzymes that need to be moved and separated by a special gel electrophoresis. While being a difficult process, it allows a relatively reproducible fractionation of an entire bacterial genome on a single gel.

Ribotyping: Ribosomal RNA (rRNA) is relatively well conserved within the species. Bacteriologists use probes on rRNA to identify the phylogenetic position of bacteria. It has been suggested that this tool may be useful in identifying the epidemiology and species differentiation of leptospira. Taking MAT/culture as the gold standard, Thaipadungpanit et al. [25] compared the diagnostic specificity and sensitivity of detection of genomic 16 s rRNA and lipL32 gene in 133 cases of leptospirosis (plus 133 controls). The diagnostic sensitivity was low with both tests, but was better in the 16 s rRNA assay (53 vs. 46%); specificity was high, but lower with 16 s rRNA (90 vs. 93%). The advantage of these tests compared to MAT is that detection of genomic material can be done at a very early stage of the illness without having to wait for antibody development. In Sri Lanka, Agampodi et al. [26] used quantitative PCR to amplify 16 s rRNA, and found that sensitivity was much better when serum was used as the source than whole blood (51 vs 18%). Quantitative leptospiraemia correlated with myocarditis, renal failure and multi organ failure. Furthermore, sensitivity of PCR was not affected by the duration of illness.

DNA sequencing: Sequencing nucleic acids at a particular genetic locus allows to identify interspecies differences and genetically classify different serovars. This is a laborious and expensive technique.

In an overall analysis of diagnostic tests for leptospirosis, the trend now is to find a test that will yield good results as early as possible in the disease process. Culture and MAT, though considered to be the gold standard, are clearly unsuitable in this regard as they are cumbersome and time consuming. Of the serological tests, several studies have indicated the possibility of utilizing antibodies against OMPs of pathogenic leptospira species for early diagnosis with good sensitivity and specificity. Most of these tests utilize IgM antibodies though some utilize IgG antibodies. However, the disadvantage is that the antigen against which the antibodies are developed may not be conserved among all pathogenic leptospirosis serovars. If that is the case then tests developed for circulating serovars in one locality may not be applicable to others. However, many proteins that have been mentioned above seem to be conserved across the pathogenic species. MAT, despite being the “gold standard” has its own problems. It requires the continuous maintenance of live leptospira antigens in a panel of different serovars. If the standard panel does not contain a locally prevalent serovar, again the diagnosis may be missed.

In most previous studies of new diagnostic tests, comparison has been made with MAT as the reference standard. The validity of comparing new immunodiagnostics with MAT as the “gold standard” has been debated [21], for the reasons mentioned earlier. Bayesian latent class modeling has been suggested over traditional gold standard analysis when evaluating new immunological diagnostic tests. The role of genetic testing has come to the fore recently mainly because of better sensitivity in early disease compared to MAT. Theoretically, genomic antigen detection would allow better and faster diagnosis, but these methods are not widely available, and are likely to be costly.

### Developing a vaccine for leptospirosis

There is currently no widely used vaccine for leptospirosis. The first vaccine introduced for leptospirosis was a killed whole cell vaccine that consisted of formalin-killed leptospires [27]. Various studies report the duration of efficacy of whole cell vaccines to be between 6 months to 7 years. However, in most studies, the duration of protection was at best 3 years [27]. The problem with this vaccine is that its serovar specific [27]. The monovalent vaccine did not protect against infection by other serovars and therefore its protection is dependent on the locally isolated serovars. This fact, plus its side effects, has led to other options being explored in vaccine designing.

Leptospiral lipopolysacharides (LPS) are an area of interest for vaccine developers. However, immunity generated by these antigens was also considered to be serovar specific. Some success with LPS vaccines has been demonstrated in animal models.

Protein antigens are a mainstay of the current drive to develop a leptospirosis vaccine. The discovery of outer membrane proteins of leptospira that were common or conserved in pathogenic species has generated interest among immunologists and vaccinologists in developing a polyvalent vaccine that is effective against different pathogenic species with minimum side effects. Subunit vaccines cause less side effects than whole cell vaccines. The proteins of interest are: Omp L1 (transmembrane protein), LipL41 (outer membrane protein), LipL32/Hap-1, Leptospiral immunoglobulin-like proteins [28] and LemA [29]. Seixas and colleagues [30] evaluated the potential of using LipL32 with various vaccine platforms to induce immunity in an animal model (rBCG vaccine, DNA vaccine and a subunit vaccine). The protein was immunogenic and the subunit vaccine gave the highest antibody titres. They further demonstrated that anti LipL32 inhibited leptospira growth in vitro.

The leptospiral immunoglobulin-like proteins consist of three proteins LigA, LigB and LigC. LigA and B have been to shown to have immunogenic potential in animal models [31]. LigB is universally present in all pathogenic leptospira serovars and therefore carries the best hope for being an immunogenic component in a recombinant universal leptospirosis vaccine [32]. Cao and colleagues [33] developed a fusion recombinant protein of two immunogenic proteins (immunoglobulin like proteins and LipL32) and the combined product (in various combinations) had good protective efficacy in a hamster model. The authors also noted that using LipL32 alone was not as successful as using the combined protein. However in a subsequent paper, a different group of investigators reported that when LipL32 is combined with B subunit of *E. coli* heat liable enterotoxin, it evoked a significant immunoprotective effect [34].

With sequencing of entire genomes of some pathogenic leptospira species, the possibility of isolating sequences that might code for membrane proteins that are potential candidates for vaccine development has opened up. Such areas can be recognized by scanning the entire genome with computer generated algorithms; identified genes can be cloned and their proteins purified to check for antigenicity [35-37]. This is a very complex process, but has immense potential for future vaccine development. Such putative protein products have been purified and tried in animal models with some promising results.

Overall, despite the advances in biotechnology, the only usable efficacious vaccine for leptospirosis to date are the whole cell inactivated vaccines. Vaccines based on recombinant membrane proteins have only been tried out in animals with limited success. The disadvantage of whole cell vaccines is that they are serovar specific (polyvalent vaccines can be made by using several serovars in one vaccine) and therefore can be used in a geographically restricted area. Nonetheless, given the limited progression on developing a universally useful vaccine active against all pathogenic serovars, the most cost effective measure for a developing country is to work on a locally effective killed whole cell vaccine.

### Molecular markers of severe leptospirosis

Leptospirosis is a disease with a wide spectrum of manifestations. Only a minority of infected people will develop severe disease with multi-organ failure. This severe disease is seen with certain serovars but not all individuals infected with a particular serovar will develop severe disease. To make matters complex, the classification of serovars is a cumbersome process that is dependent on detailed immunological phenotyping. It does not relate with leptospira species categorization which is based on DNA analysis. While serovar diagnosis is relatively freely available, DNA based species categorization is only available in reference genetic laboratories. This creates a barrier in correlating clinical features with the infecting species.

Why certain infections in some people lead to severe disease, while others have a mild illness, is an unresolved mystery. Current thinking is that both pathogen related (infecting serovar/species, innoculum size) and host factors (immunological response) contribute to this heterogeneity. However, if certain markers of severe disease can be identified either in the host or the pathogen, it will be of great help in predicting severe disease.

One particular compound that has been of interest in this regard is nitric oxide. It is known that in a state of inflammation, release of inflammatory cytokines (TNF-α, IL-1,6) activate inducible nitric oxide synthatase (iNOS) to produce NO which is bacteriostatic. NO is metabolized to nitrite, which has a short half-life in blood, and then to nitrate. Estimation of NO activity can be made by measuring nitrites, nitrates or both. There is limited evidence as to which metabolite most accurately reflects NO activity in response to severe infection. Nitrite is likely to be more specific, as it has a short half life, and is less affected by renal function. It was hypothesized that in severe leptospirosis the level of NO may be elevated. This hypothesis was confirmed by two separate studies six years apart, where serum NO levels were shown to be raised in patients with symptomatic leptospirosis [38,39]. However, a paradox in serum nitrate concentrations (a surrogate marker of NO) has been demonstrated in malaria where people with severe malaria actually had paradoxically low total NOx (nitrite and nitrate) levels when it was corrected for serum creatinine. In severe leptospirosis, since there is renal impairment, it is possible that the raised NO level may not reflect increased synthetic activity but reduced clearance of NO via the kidneys. In fact a recently published paper by Kalugalage et al. [40] demonstrated that, as in malaria, the corrected NO concentration (corrected for renal impairment) in patients with severe leptospirosis is actually lower than in non-leptospirosis fever patients and patients with mild leptospirosis. The pathophysiological basis for this phenomenon remains elusive. Whether low NO levels contribute to pathogenesis of severe disease or whether it is a result of severe disease and acute kidney injury is unclear. Interestingly, there is an animal study by Pretre et al. [41] where infected mice and hamsters showed increased iNOS mRNA and protein in kidneys compared to controls. Giving the animals 4-aminopyridine, which is a iNOS inhibitor, caused faster deterioration. NO is one of the mediators which drives oxidative stress, and, like in many other diseases, oxidative stress is likely to play a role in tissue and organ damage in leptospirosis, although currently evidence on this is limited.

Studies of immunochemical markers have shown that both cell mediated as well as humoral immunity are activated in severe leptospirosis. De Fost et al. [42], in an analysis of 44 Thai patients with definite or suspected leptospirosis, showed that markers of cell mediated immune activity was raised compared to healthy controls (Interferon [IFN]-gamma-inducible protein-10, granzyme B, monokine induced by IFN-gamma).

Proteomics seem to be a promising tool to study the inflammatory response in acute leptospirosis. The genomic sequences, despite being highly conserved among members of a species, do not show the functional status of a living cell (as genes are selectively turned on and off). Study of mRNA, though theoretically better to analyze gene expression, has many technical difficulties in practice (they are rapidly degraded, and not all mRNA are translated to proteins). In the light of these findings, the best way to assess the functional status of a cell is to assess its protein profile. However assessment of such profiles is complex, as these profiles change with time and from cell to cell depending on gene activation. Mass scale analysis of proteins in leptospirosis patients with severe disease has enabled identification of proteins that are differentially expressed in severe disease. Such identified proteins can be targets for further studies on pathogenesis and vaccine development [43,44]. In the most recently published study on proteomic analysis of serum of leptospirosis patients (compared to controls with malaria and healthy volunteers) Srivastava and colleagues [45] demonstrated several differentially expressed proteins in leptospirosis patients that were not previously associated with the disease pathogenesis. Therefore this is a rapidly evolving field.

Whether certain hosts of a particular genetic makeup have increased vulnerability to leptospirosis is of interest. A study by Fialho et al. [46] compared victims of leptospirosis with healthy controls for HLA alleles and genetic polymorphisms in the cytokine genes. Significant associations were found for certain alleles of HLA-A,B loci plus several HLA haplotypes. Polymorphisms in IL-4 and IL-4Rα genes were also significantly associated with a past history of leptospirosis. However, these findings have not been confirmed in larger population samples. Other studies have assessed different mediators of sepsis and cytokines in relation to severe leptospirosis. These mediators include human serum mannose binding lectin (which identifies pathogens activating the immune system) [47], soluble ST2 receptors, long pentraxin PTX3, copeptin and platelet activating factor acetylhydrolase (limited studies in animal models).

Membrane bound ST2 (mST2) is a negative regulator of toll like receptors (which is an important component of innate immunity). sST2 (soluble ST2) inhibits signaling via mST2. In an observational study in 68 severe leptospirosis patients, Wagenaar et al. [48] demonstrated that sST2 levels, cytokines IL-6, IL-8, and IL-10 were elevated in all patients. However sST2 levels had a significant association with any bleeding manifestation and severe bleeding. It also had a significant association with mortality (OR 2.4; 95% CI: 1.0-5.8). Interleukins 6 and 8 also showed a significant association with mortality but not with bleeding. In another study of assessing biomarkers of clinically severe leptospirosis, Wagenaar et al. [49] have shown that PTX3, a long pentraxin (pentraxins are a super family of large multimeric proteins that are thought play an important role in innate immunity and adjusting immune response) was elevated in leptospirosis and showed a significant association with mortality and disease severity. C-reactive protein is a structurally related protein (short pentraxin) but it did not show such a correlation with disease severity or death. In this study, both IL-6 and 8 were also shown to have a significant association with mortality. On the same cohort of patients, authors have also shown that copeptin (a stable peptide of arginine vasopressin precursor that is released in increased amounts in sepsis) levels were elevated in patients with severe leptospirosis and elevated levels were significantly associated with high mortality [50].

The study of biomarkers for severe disease has become more complex with recent genome wide studies in leptospira genome. Comparative analysis of saprophytic and pathogenic leptospira has shown that nearly 900 genes in pathogenic strains may be contributing to the pathogenicity of disease [51]. The functions of most of these genes are unknown and the known proteins which are thought to be of functional significance cannot explain all the virulence mechanisms of the organism. To make matters more complicated, it has been demonstrated that some of these genes are differentially regulated depending on the ambient conditions (temperature, osmolarity and iron levels). Mutation analysis systems have shown that some genes have definite roles in pathogenesis (as mutations in these genes attenuate virulence) and these include OmpA-family protein, Loa 22 and several other proteins [51].

Identified areas for further research in this fast developing field are; a) serial measurement of NOx levels in patients with leptospirosis to identify its use as a predictor of severity, b) further analysis of NOx with correction for creatinine released from muscle, c) further exploration of the role of oxidative stress in tissue and organ damage, d) use of cytokines as predictors of disease severity and e) proteomic analysis of sera of severe leptospirosis, mild leptospirosis, non leptospirosis fever patients (and healthy controls) on admission and serially to identify differentially expressed proteins that can be potential severity markers.

## Conclusions

The ideal diagnostic test for leptospirosis should give a positive result as early as possible, should have good sensitivity and specificity plus be cost effective. MAT which is the presumed “gold standard” for leptospirosis is probably unsuitable for routine diagnosis due to its high false negative readings in early disease, lack of specificity for acute infection, and the cumbersomeness of the process. Other immunological methods such as immunochromatography and IgM ELISA have shown promise with early diagnosis and good sensitivities and specificities compared to MAT. Given the fact that MAT is may not be the ideal gold standard, Bayesian latent class models have shown that the sensitivities and specificities of these other tests may be higher than expected. Genomic diagnostics offer another exciting diagnostic possibility in early disease. However, the yields of these tests are low and they also need expensive equipment that is not freely available. Their use is currently limited to research and genotypic analysis.

The quest for a successful vaccine continues. The most efficient vaccines to-date are the whole cell killed vaccines which were also the earliest vaccines developed against leptospirosis. The disadvantage of these are that they are either monovalent or offers protection to a few locally circulating serovars. Research on subunit vaccines which offers universal protection against all pathogenic leptospira have not shown promising results despite having identified several proteins that are conserved among all pathogenic leptospira identified to-date.

Clinical features are not very good predictors of potential disease severity and therefore much of the recent focus in leptospirosis research is on identification of biomarkers that will predict severe disease in patients. Immunological studies have evaluated the role of cytokines such as IFN-γ, IL-6 and IL-8 in leptospirosis. Non specific activation of other cytokines such as TNF-α and IL-1 can increase the oxidative stress and free radicals. These may induce nitric oxide synthase activity resulting in higher total nitrite levels and overall reduced antioxidant capacity. They may have value as severity predictors. Genetic heterogeneity of HLA alleles, cytokine genes and proteomics of host and genomics of the pathogen are new ongoing avenues in research that might shed light in to having robust predictors for severe disease in future.
